# Supplementary material for: XENOFOOD—An Autoclaved Feed Supplement Containing Autoclavable Antimicrobial Peptides—Exerts Anticoccidial GI Activity, and Causes Bursa Enlargement, but Has No Detectable Harmful Effects in Broiler Cockerels despite In Vitro Detectable Cytotoxicity on LHM Cells
Source: Pathogens. 2023 Mar 14;12(3):458. doi: 10.3390/pathogens12030458 (PMC10059668; doi:10.3390/pathogens12030458)
Supplement: Supplementary file 1 [file pathogens-12-00458-s001.zip › pathogens-2207588-supplementary.pdf]

Table S1. The scoring system published by Amin et al. (2012) ˙[47] was applied to assess the degree of LMH monolayer destruction.

		SCORES (Cell Layer Color Intensity)				
**Score**		**0**	**1**	**2**	**3**	**4**
0	intact monolayer	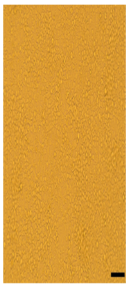	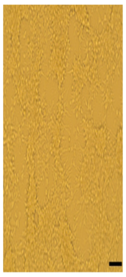	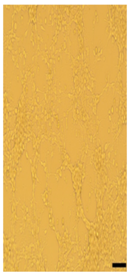	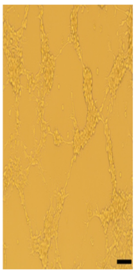	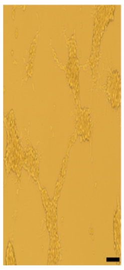
1	up to 25% of the monolayer destructed					
2	25–50% of the monolayer destructed					
3	50–75% of the monolayer destructed					
4	more than 75% of the monolayer destructed					

[1] Scoring system for the degree of LMH monolayer destruction Abbreviations: EPB = entomopathogenic bacteria, CFCM = cell-free conditioned culture media.

Table S2. Stocks solutions and dilutions, and dilutions of EMA and EMC, CFCMs.

Dilutions	EMA 80%	EMC 60%
1:2.5	32%	24%
1:5	16%	12%
1:7.5	10.63%	7.97%
1:10	8%	6%
1:25	3.2%	2.4%
1:50	1.6%	1.2%
1:75	1.063%	0.797%
1:100	0.8%	0.6%

Table S3. Results (Scores) of Experiment 2.

Sample		24 h	48 h	72 h	96 h
Original (unchanged) media in which the cell layer had developed RPMI1640 + 10%FKS + 0.5% AB	A	0	0	0	0
	B	0	0	0	0
Fresh media (original media replaced by fresh RPMI1640 + 10%FKS + 0.5% AB)	A	0	0	0	0
	B	0	0	0	0
Original media replaced by EMA 80% 1:2.5	A	4	4	4	4
	B	4	4	4	4
EMA 80% 1:5	A	3	4	4	4
	B	3	3	4	4
EMA 80% 1:7.5	A	2	2	4	4
	B	2	2	3	3
EMA 80% 1:10	A	1	2	2	2
	B	1	2	2	2
EMA 80% 1:25	A	1	1	0	0
	B	1	1	0	0
EMA 80% 1:50	A	1	1	0	0
	B	1	1	1	1

EMA 80% 1:75	A	1	1	0	0
	B	1	1	1	0
EMA 80% 1:100	A	1	1	0	0
	B	1	1	1	0
EMC 60% 1:2.5	A	2	3	4	4
	B	2	3	4	4
EMC 60% 1:5	A	1	1	2	2
	B	1	1	2	2
EMC 60% 1:7.5	A	1	1	2	2
	B	1	1	2	2
EMC 60% 1:10	A	1	1	2	2
	B	1	1	2	2
EMC 60% 1:25	A	1	1	0	0
	B	1	1	0	0
EMC 60% 1:50	A	1	1	0	0
	B	1	1	1	0
EMC 60% 1:75	A	1	1	0	0
	B	1	1	0	0
EMC 60% 1:100	A	0	0	0	0
	B	0	0	0	0

Table S4. Composition of experimental diets.

	**Starter**	**Grower**	**Finisher**
wheat	48.0	49.9	51.1
maize	5.0	5.0	6.0
ext. soybean meal	32.7	27.9	23.0
ext. sunflower meal	2.0	3.0	4.0
sunflower oil	4.5	6.0	7.0
DDGS	3.0	4.0	5.0
limestone	1.63	1.37	1.32
MCP	1.67	1.45	1.32
salt	0.20	0.19	0.18
NaHCO3	0.30	0.30	0.30
Choline chloride	0.025	0.025	0.025
L-Lysine HCL	0.37	0.29	0.27
DL-Methionine	0.34	0.27	0.22
L-Threonine	0.16	0.12	0.1
vitamin premix	0.05	0.05	0.04
mineral premix	0.1	0.1	0.1

Table S5. Nutrient content of the experimental diets (g/kg).

	**Starter**	**Grower**	**Finisher**
AMEn (MJ/kg)	11.99	12.54	12.93
D.M.	881.56	882.80	883.87
C. protein	235.39	220.44	205.62
C. fat	62.97	78.61	89.53
C. fiber	38.86	39.10	39.32
LYS	14.31	12.59	11.31
MET	6.79	5.96	5.31
MET + CYS	10.69	9.68	8.86
THR	9.82	8.89	8.14
TRP	2.85	2.67	2.48

Dig. LYS	12.74	11.16	10.01
Dig. MET	6.28	5.45	4.82
Dig. M+C	9.44	8.49	7.71
Dig. THR	8.32	7.43	6.72
Dig. TRP	2.40	2.22	2.03
Na	1.75	1.74	1.73
Cl	2.24	2.06	1.99
K	9.82	9.13	8.42
Mg	1.79	1.74	1.70
Ca	10.65	9.18	8.66
total P	7.93	7.41	7.09
Dig. P	5.01	4.52	4.21
